# Synthesis, spectral, thermal, crystal structure, Hirschfeld analysis of [*bis*(triamine)Cadimium(II)][Cadimum(IV)tetra-bromide] complexes and their thermolysis to CdO nanoparticles

**DOI:** 10.1186/s13065-016-0183-y

**Published:** 2016-06-13

**Authors:** Ismail Warad, Fuad Al-Rimawi, Assem Barakat, Saida Affouneh, Naveen Shivalingegowda, Neartur Krishnappagowda Lokanath, Ibrahim M. Abu-Reidah

**Affiliations:** Department of Chemistry, Science College, An-Najah National University, P.O. Box 7, Nablus, Palestine; Chemistry Department, Faculty of Science and Technology, Al-Quds University, P.O. Box 20002, Al-Quds, Palestine; Department of Chemistry, College of Science, King Saud University, P. O. Box 2455, Riyadh, 11451 Saudi Arabia; Department of Chemistry, Faculty of Science, Alexandria University, Ibrahimia, P.O. Box 426, Alexandria, 21321 Egypt; Elearning Center, An-Najah National University, P.O. Box 7, Nablus, Palestine; Institution of Excellence, VijnanaBhavan, University of Mysore, Manasagangotri, Mysore, 570 006 India; Department of Studies in Physics, University of Mysore, Manasagangotri, Mysore, 570 006 India

**Keywords:** Cadmium(II) complexes, Triamine, XRD, CdO nanoparticles

## Abstract

**Background:**

The coordination chemistry of cadmium(II) with diamine ligands is of particular interest. The most common structure around cadmium(II) center in their complexes is tetrahedral, that is due the octet rule obeyed. Nevertheless, five and six-coordinated complexes are also well known. Now a day, many cadmium(II) complexes with chelate ligands were synthesized for their structural or applications properties. Antibacterial activities and DNA binding affinity of this class of cadmium complexes have attracted considerable interest.

**Results:**

Cadmium(II) complexes in dicationic form with general formula [Cd(dien)_2_]CdBr_4_ complex **1** (dien = diethylenetriamine) and [Cd(dipn)_2_]CdBr_4_ complex **2** (dipn = diproylenetriamine) were prepared and elucidated there chemical structures by elemental analysis, UV–Vis, IR, TG and NMR, additionally complex **1** structure was solved by X-ray diffraction study. The Cd(II) cation is located in a slightly distorted octahedral geometry while Cd(IV) anion is in tetrahedral geometry. High stability of the synthesized complexes confirmed by TG. Thermolysis of complex **1** revealed the formation of pure cubic nanoparticles CdO which was deduced by spectral analysis. The average size of CdO nanoparticles was found to be ~60 nm.

**Conclusions:**

Two new Cd(II) complexes of general formula [Cd(N_3_)_2_]CdBr_4_ were made available. The structure of [Cd(dien)_2_]CdBr_4_ was confirmed by X-ray diffraction. Thermal, electro and spectral analysis were also investigated in this study. The direct thermolysis of such complexes formed a cubic CdO regular spherical nanoparticle with the ~60 nm average particle size.

**Graphical abstract:**
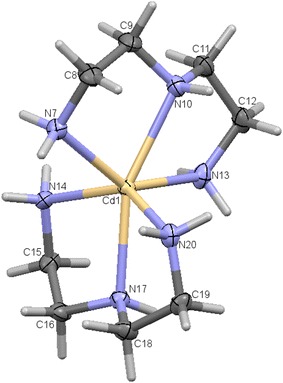
ORTEP for the complex **1**

## Background

Cadmium(II) complexes with polydentate nitrogen ligands, mainly polyamines, have been studied for some time either because of their structural properties [1, 2] or their applications [3–7]. The synthesis and characterization of triamine complexes of transition and non-transition metals are of interest as they can potentially exist in three isomeric forms, i.e. mer and fac [8, 9]. The shape of cadmium(II) halide complex anions depending on the number of hydrogen bonds and the cations species [2–5]. There are variable shapes of the complex anions such as tetrahedral [10, 11], two-dimensional layered structures [12], and complex chain structures [13–15]. Cadmium complexes have attracted considerable interest due to pharmacological importance including anti-microbial agents [4], DNA binding affinity [3], and anticancer activities [5–7, 16, 17].

The design and development of novel functional materials utilizing non-covalent interactions in complexes have attracted considerable attention [17–20]. Various weak dispersive interactions, such as hydrogen bonding and other weak interactions involving π-cloud of the aromatic ring represents the backbone of self-assembly process to stabilize the crystals [22]. Hydrogen bonding interactions are the most reliable and widely used in building multi-dimensional supramolecular structures [21–23].

In the last decade, spherical shape metal oxide nanoparticles [24] composed of a mixed-ligand dinuclear and mononuclear cadmium(II) complexes building blocks [25–28]. We reported the synthesis and characterization of two now dicationic cadmium(II) complexes with general formula [Cd(N_3_)_2_]CdBr_4_. Complex **1** used as building block for preparation the CdO nanoparticles by direct open atmosphere thermolysis process.

## Results and discussion

### Synthesis of the desired complexes

**Scheme 1:**
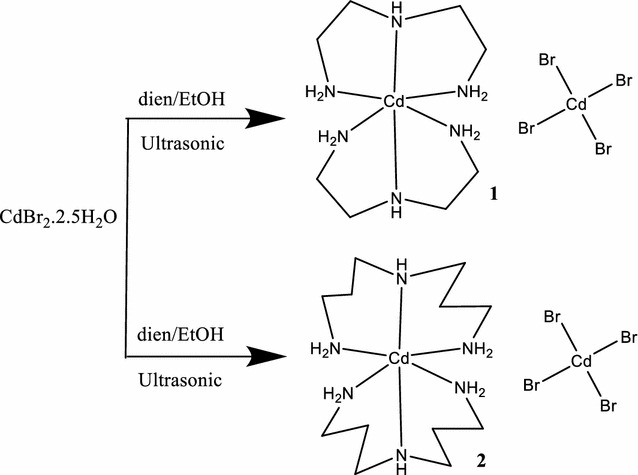
Synthesis of the desired complexes

Two new dicationic Cd(II) complexes with general formula [Cd(N_3_)_2_]CdBr_4_ have been prepared by mixing of excess of the tridentate free ligands with CdBr_2_•2.5H_2_O in EtOH under open ultrasonic atmosphere. The dicationc Cd(II) complexes were prepared in very good yield without side products, as seen in Scheme 1.

The X-ray single crystal diffraction technique used to confirm the structure of the target complex **1** and other spectral analysis including elemental analysis, IR, UV–vis, TG/DTA, CV and NMR. The isolated complexes are stable in air, soluble only in water, DMF and DMSO. The dicationc natural was supported by high water solubility (0.02 g/ml at RT) and molar conductance (ʌ_M_ = 190 Ω^−1^cm^2^ mol^−1^ of 1 × 10^−3^M at RT) showed that the two complexes are electrolytic in their nature. The analytical data of the [Cd(dien)_2_]CdBr_4_ desired complex consisted with XRD analysis data. The TG-residue product of complex **1** revealed the formation of CdO cubic nanoparticle [23]. The genital heating with fixed heat of rate as well as the N-tridentate ligands may play the critical role in de-structure of the desired complexes to CdO nanoparticles.

### X-ray crystal structure of complex **1**

**Fig. 1:**
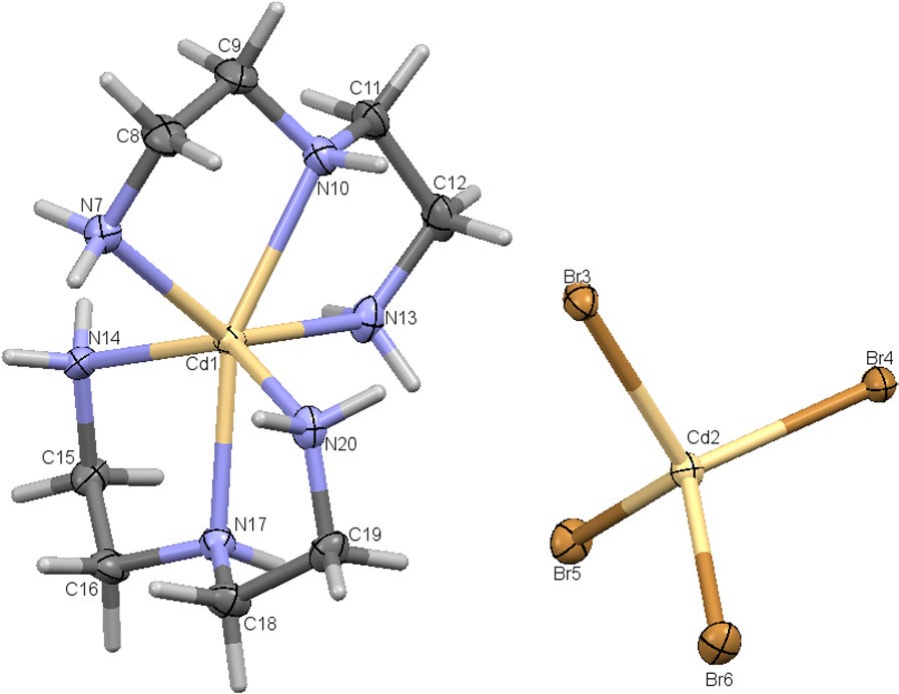
ORTEP of the complex **1** with atom labelling. Thermal ellipsoids are drawn at the 50 % probability level

**Fig. 2:**
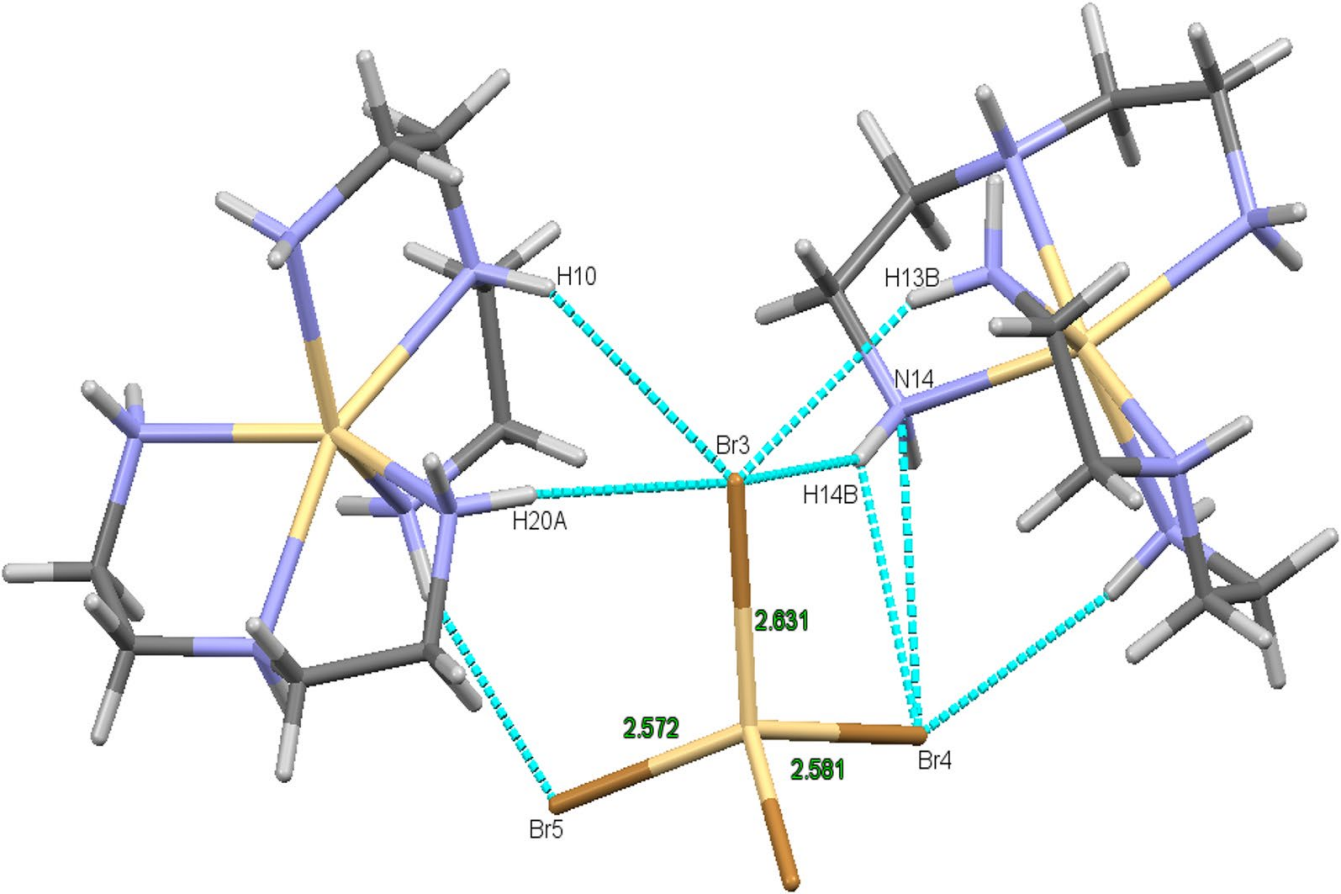
Elongation of bond length of Br3 atom due to hydrogen bonding. The *dotted lines* indicate hydrogen bonds

**Fig. 3:**
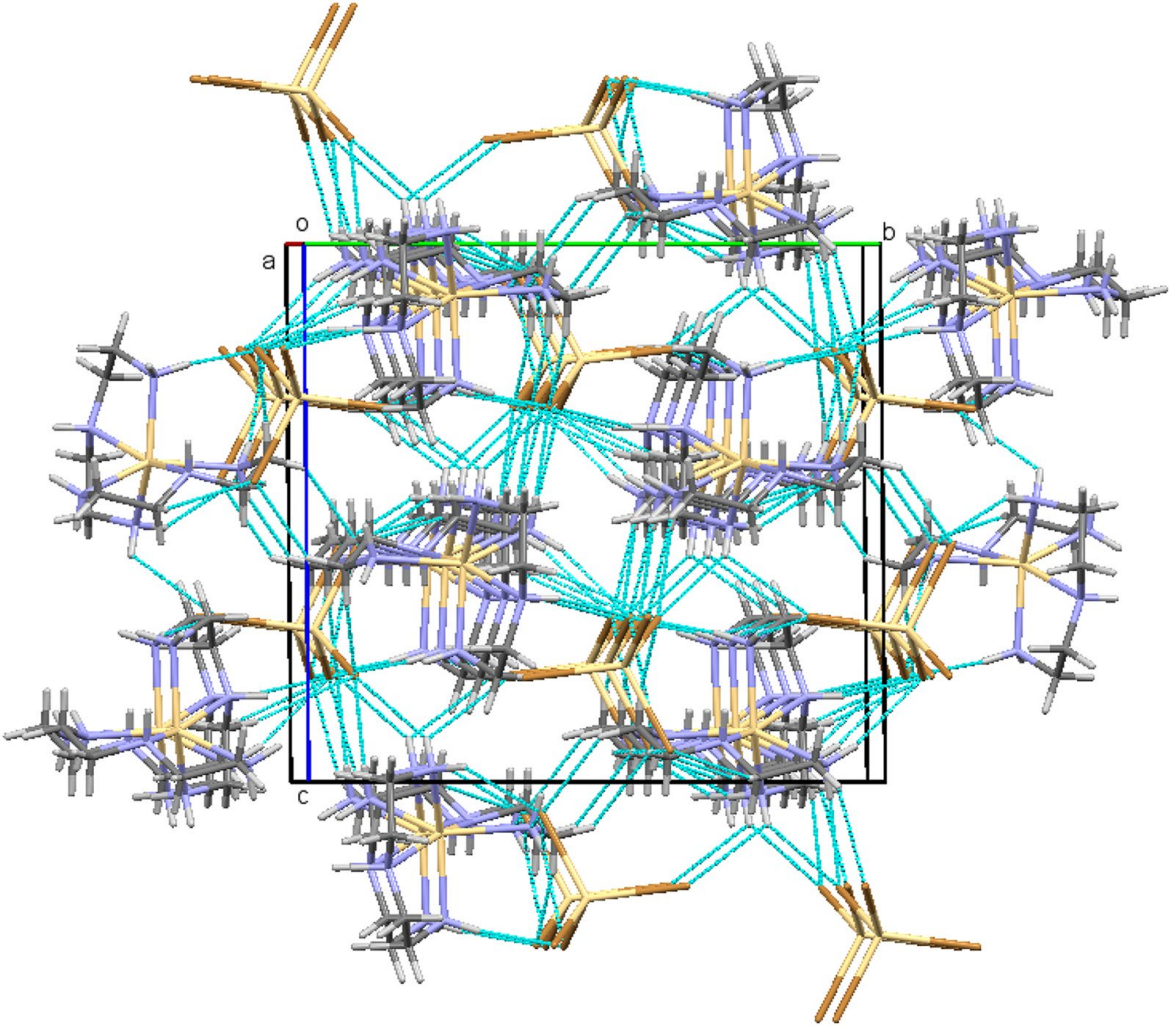
A crystal packing of complex **1** exhibiting layered stacking when viewed (perspective) along the crystallographic *a* axis. The *dotted lines* indicate hydrogen bonds

An asymmetric unit cell consists of two Cd^2+^ ions of which one is a cation and the other counter ion, two dien fully coordinated to the Cadmium cation center. An N6 coordinated complex is formed. The Cd(II) cation are located in a slightly distorted octahedral geometry while Cd(IV) counter anion are in tetrahedral geometry seen in Fig. 1. The bond length between the Cd(IV) anions and the bromine atoms are in the expected range except for the elongation of Br3 atom which is actively involved in the hydrogen bonding as seen in Fig. 2. This type of hydrogen bonding helps in the better stabilization of the crystal structure. A study of torsion angles, asymmetric parameters and least-square plane calculations reveals that one of the four five membered ring the ring adopts an envelope conformation with the atoms N10 and N13 deviating 0.230 (3) and −0.109 (3) Å respectively from the Cremer and Pople plane [29]. This is confirmed by the puckering parameters Q = 0.472 (3) Å and ϕ = 255.5 (3). The other three five membered rings adopts a twisted conformation on the bonds C8–C9, C15–C16 and C18–C19 respectively. The structure exhibits both inter and intramolecular hydrogen bonds of the N–H….Br and C—H….Br which helps in stabilizing the crystal structure [14, 15]. Packing of the molecules when viewed down along the *a* axis indicates that the molecules exhibit layered stacking and several hydrogen bonds as seen in Fig. 3. The crystal data deposited and can be retrieved via CCDC 1404033.

### IR spectrum

**Fig. 4:**
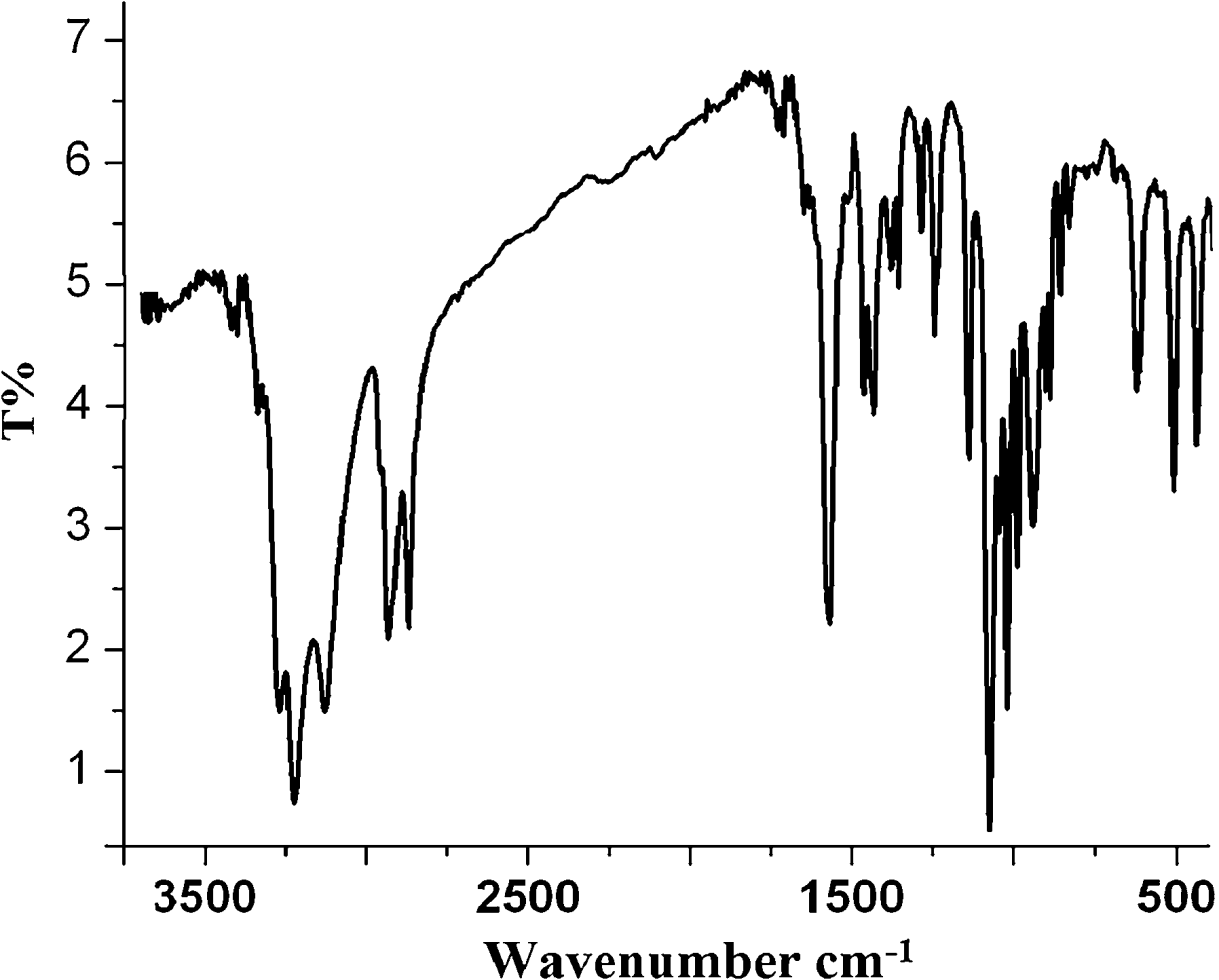
IR-KBr disk spectra of the complex **1**

The IR spectrum of complex **1** is depicted in Fig. 4. Complex **1** revealed three main characteristic absorptions peaks in the range 3180–3300, 2780–2850 and 650–450 cm^−1^, which was assigned to N–H, C-H_alkyl_ and Cd–N stretching vibrations, respectively [25–27]. No water was recorded in the structures of the complexes. The chemical shifts of N–H functional groups of dipen coordinated to the Cd(II) center in the complexes was shifted down filed by ~60 cm^−1^ compared by the free one, this support the tridentate ligand full coordination to the Cd(II) center.

### UV–Vis spectral study

**Fig. 5:**
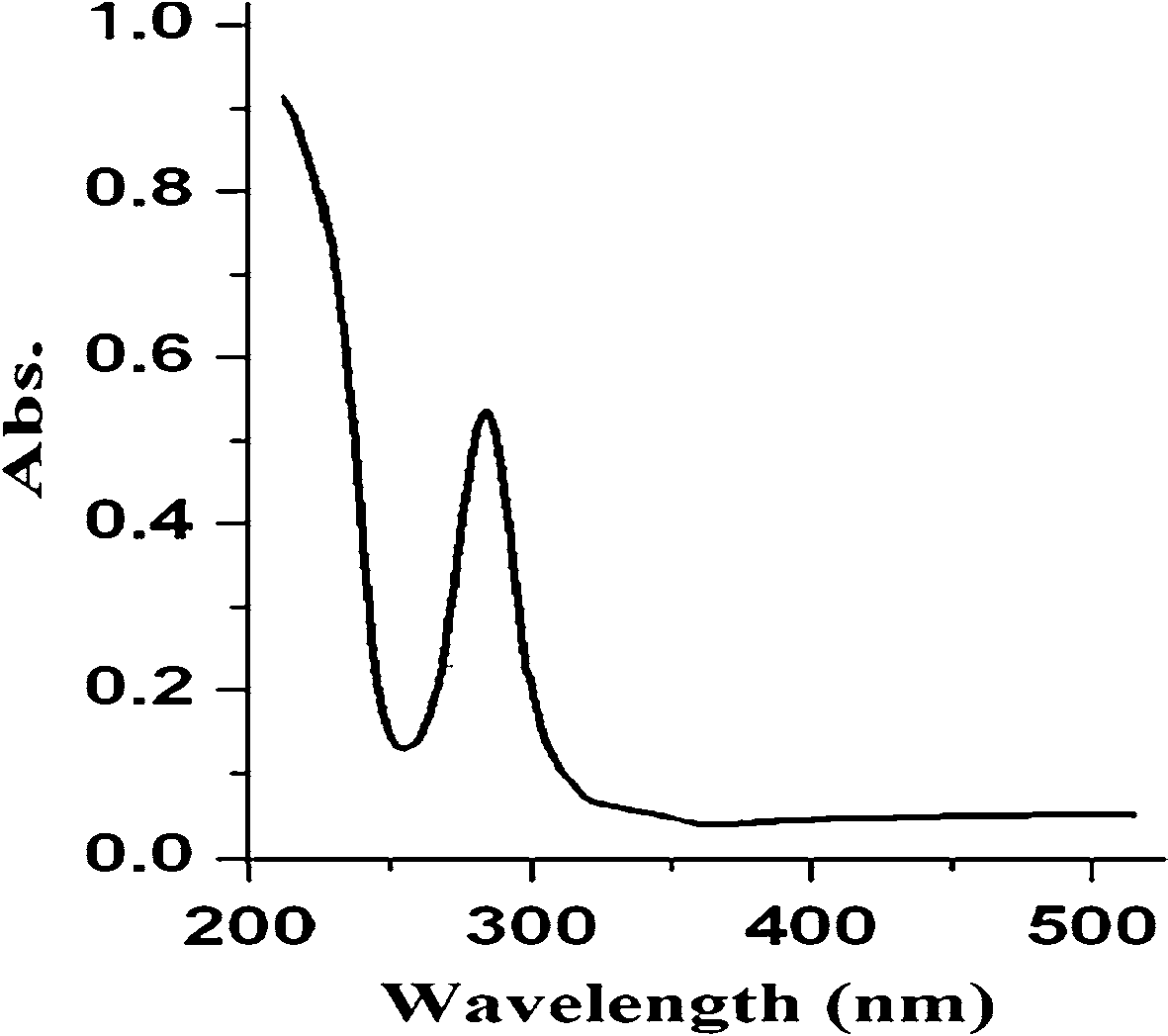
UV–Vis spectrum of the complex **1** in water at RT

The UV–Vis absorption spectra of the complex **1** and complex **2** in water solvent presented one sharp dominant bands at 270 and 280 nm respectively, no other bands were detected elsewhere, as seen in Fig. 5. The cadmium centers showed only the charge transfer transitions which can be assigned to charge transfer from the metal to ligand and vice versa (d—σ* electron transfer), no absorption resonated to π–π* electron transfer (dien and dipn ligands are saturated) or d–d transition are expected for d^10^ Cd(II) complexes [30, 31].

### NMR investigation

**Fig. 6:**
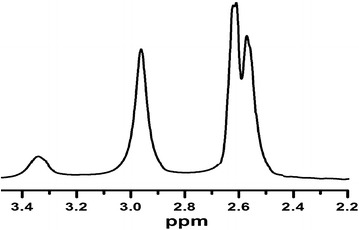
^1^H NMR spectrum of the complex **1** in DMSO at RT

The ^1^H and ^13^C{^1^H} NMR spectra of the synthesized complexes were carried out in d^6^-DMSO solvent to confirm the binding of the dien ligands to the cadmium(II) in 2–1 ration respectively. The ^1^H and ^13^C{^1^H} NMR spectra corroborate the structure of the desired complexes as well as the XRD; only three functional groups, ^1^H NMR (d^6^-DMSO): d (ppm) 2.55 and 2.62 (2 br, 16H, 8CH_2_), 2.85 (br, 8H, 4NH_2_), 3.35 (br, 2H, 2NH), signals belonging to the CH_2_CH_2_ and NH_2_ of dien ligand coordinated with CdBr_2_ were recorded, as depicted in Fig. 6.

### TG analysis

**Fig. 7:**
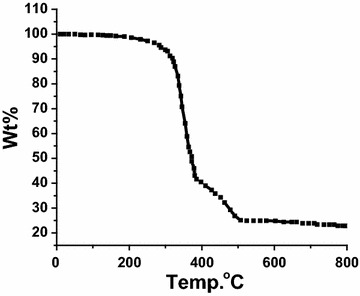
TG thermal curve of complex **1**

The TG of the complex was carried out in the range of 0–800 °C and 10 °C/min heating rate, typical thermal TG curve are given in Fig. 7 which shows that there is no coordinated or uncoordinated water in the range 0–180 °C. Also organic and inorganic contents were de-structured away (to CO_2_, NO_x_ gas product) from the Cd(II) metal center in one step decomposition in range 290–500 °C with ~80 % weight loss. The final product (20 % residue) was confirmed to be CdO by IR [32–34].

### CdO nanoparticle formed by direct thermolysis of complex **1**

The phase information and composition of the TG final residue produced through open atmosphere thermolysis of complex **1** was deduced by FT-IR, X-ray powder diffraction (XRD), EDX, SEM and TEM. The product was characterized as CdO nanoparticles.

**Fig. 8:**
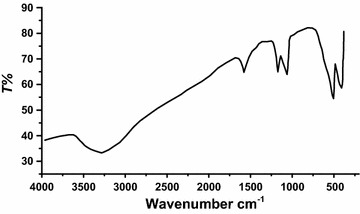
IR spectra of CdO nanoparticles produced by thermolysis of complex **1**

Figure 8 shows the IR spectrum product CdO nanoparticle, the formation of CdO nanoparticle was supported by two signs vibration at 420 and 560 cm^−1^ belongs to Cd=O bond, it could be useful in understanding the bonding between the Cd–O atoms [32]. All the other vibration assigned to the starting complexes was disappeared due to the thermal digestion of all organic contents.

**Fig. 9:**
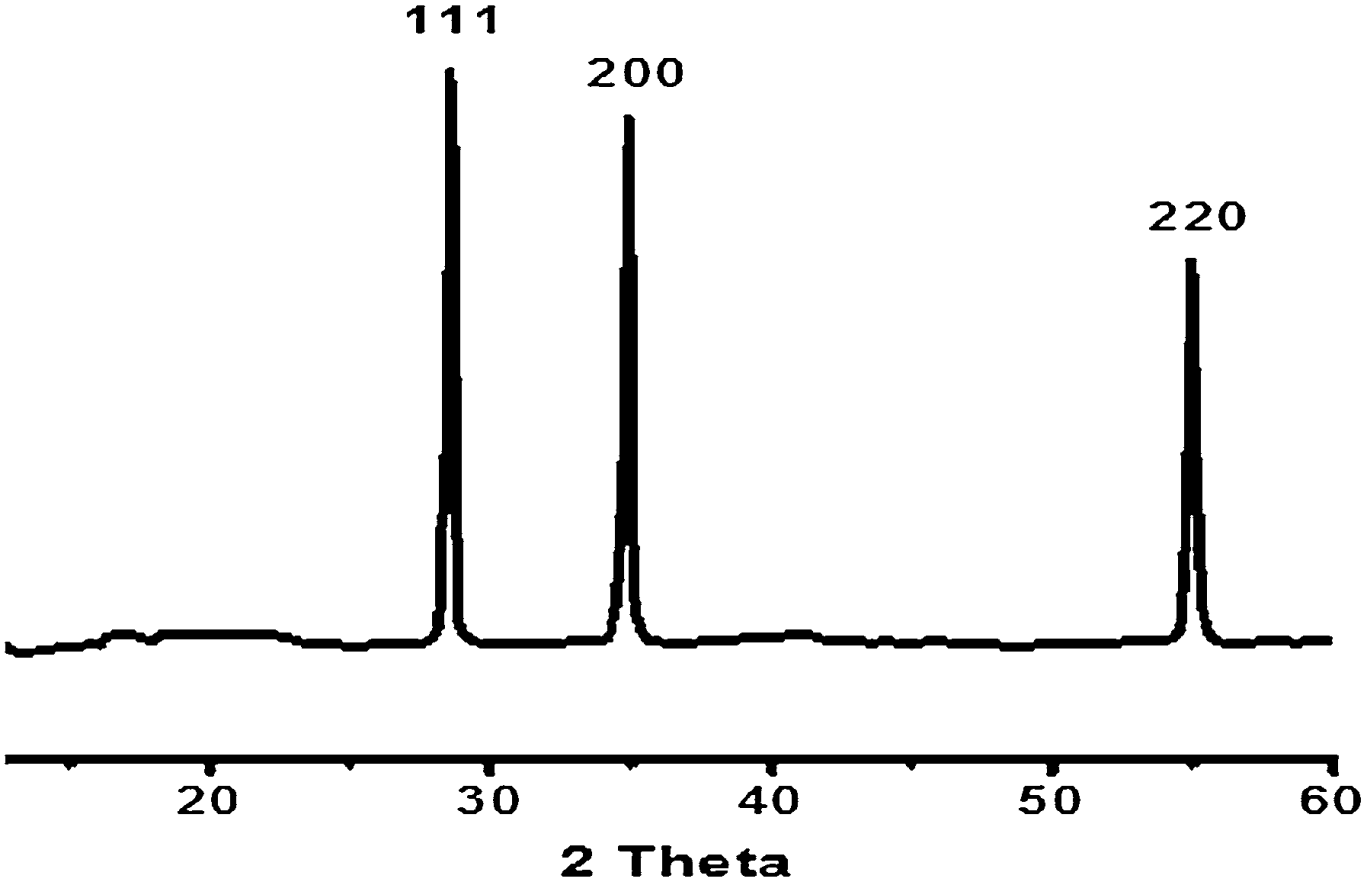
Powder XRD pattern of CdO prepared by direct thermolysis of the complex **1**

The (111), (200) and (220) reflections are closely match the reference CdO prepared with JCPDS file No. 05-0640, the formation of CdO cubic crystal nanoparticle was confirmed, see Fig. 9. The particles were found in polycrystalline structure and that the nanostructure grew in a random orientation which confirmed by sharp peaks from XRD data [32–36].

**Fig. 10:**
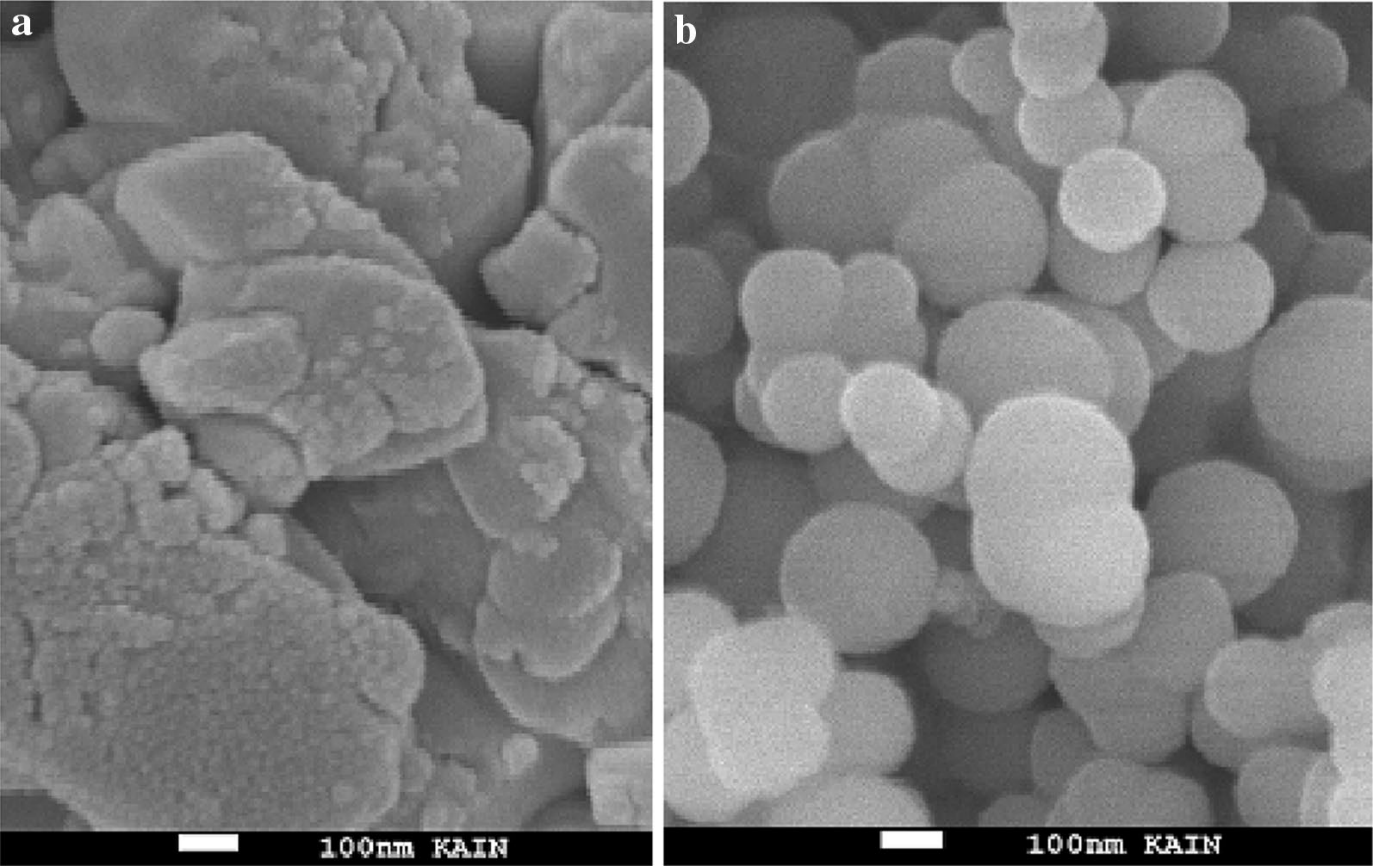
The SEM image of complex **1** **a** before and **b** after calcination to produce CdO nanoparticles

The size and morphology of these particles were determined by Scanning Electron Microscopy (SEM) before and after calcination, as seen in Fig. 10a, b, respectively. SEM image for complex **1**, particles were irregular before calcination, while after calcination regular spherical particles were collected, which confirmed that tridentate organic ligands play de-structure role during thermolysis process [30–36]. According to this micrograph, nanoparticles with less than 100 nm in diameter were produced.

**Fig. 11:**
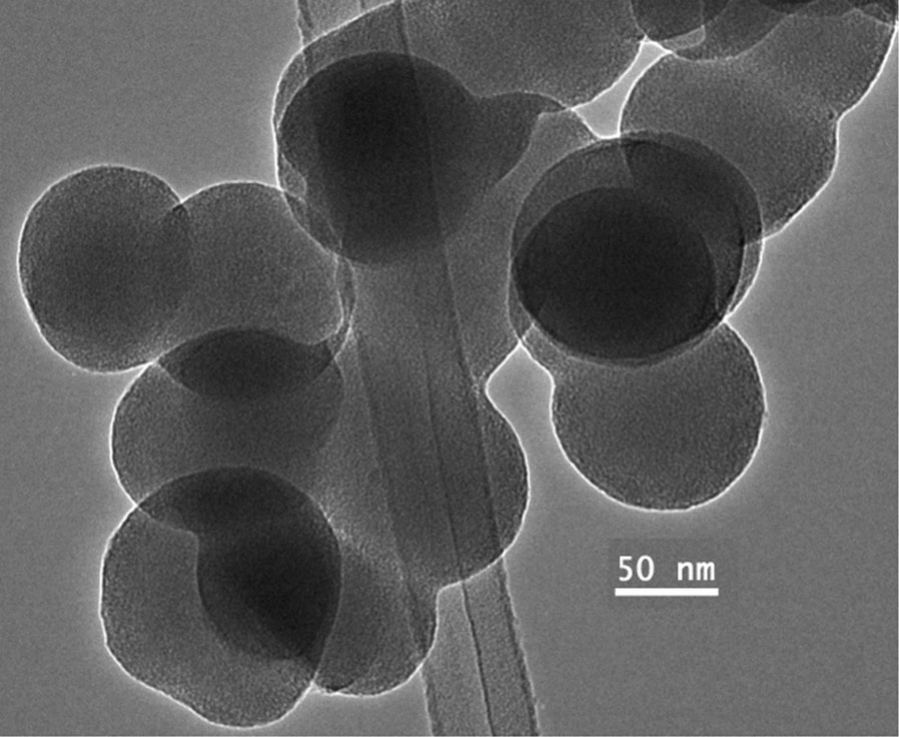
TEM image of CdO nanoparticles of an average diameter of ~60 nm

Also, TEM was carried out for the CdO nanoparticles corresponding to the same sample above was illustrated in Fig. 11. From TEM image, the average size of the nanoparticles found to be around 60 nm. The particles are spherical in shape, not unlike those reported by Dong et al. [34].

### Hirshfeld surface analysis for complex **1**

**Fig. 12:**
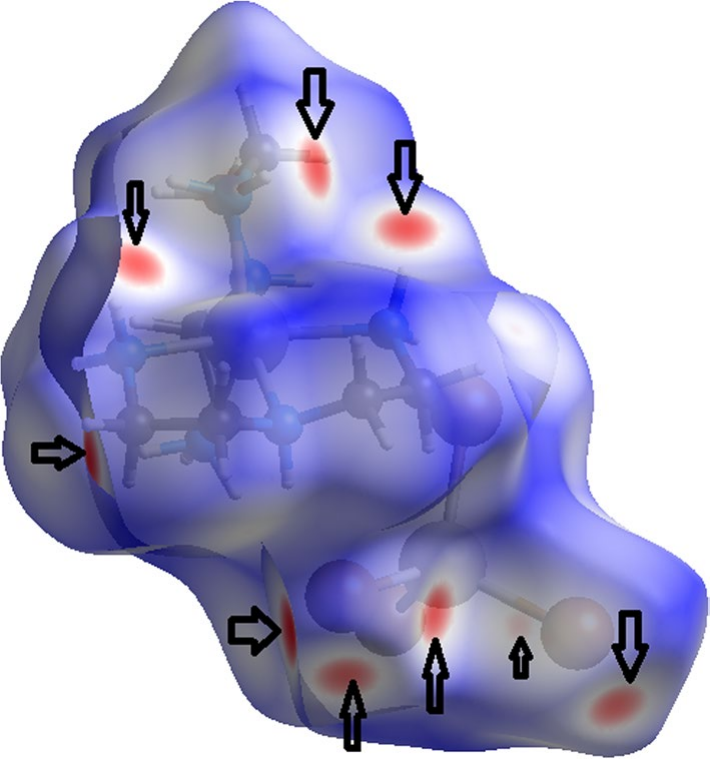
*d*_*norm*_ mapped on hirshfeld surface for visualizing the intercontacts of complex **1**

Crystal structure analysis of complex 1 using the cif file was generated by Hirshfeld Surface, to analysis the intermolecular interactions then illustrated the fingerprint map of atoms_inside_/atom_outside_ interactions of molecules. The Hirshfeld surfaces of complex **1** is displayed in Fig. 12, showing surfaces that have been mapped over a d_norm_, d_e_ and d_i_ [37, 38]. “For each point on that isosurface two distances are determined: one is d_e_ represents the distance from the point to the nearest nucleus external to the surface and second one is d_i_ represents the distance to the nearest nucleus internal to the surface. The dark-red spots on the d_norm_ surface arise as a result of the short interatomic contacts, i.e. strong hydrogen bonds, while the other intermolecular interactions appear as light-red spots [18–22]”. The surface here in this work represents the circular depressions (deep red) visible on the Hirshfeld surface indicative of strong hydrogen bonding contacts of types N–H….Br and C—H…..Br.

**Fig. 13:**
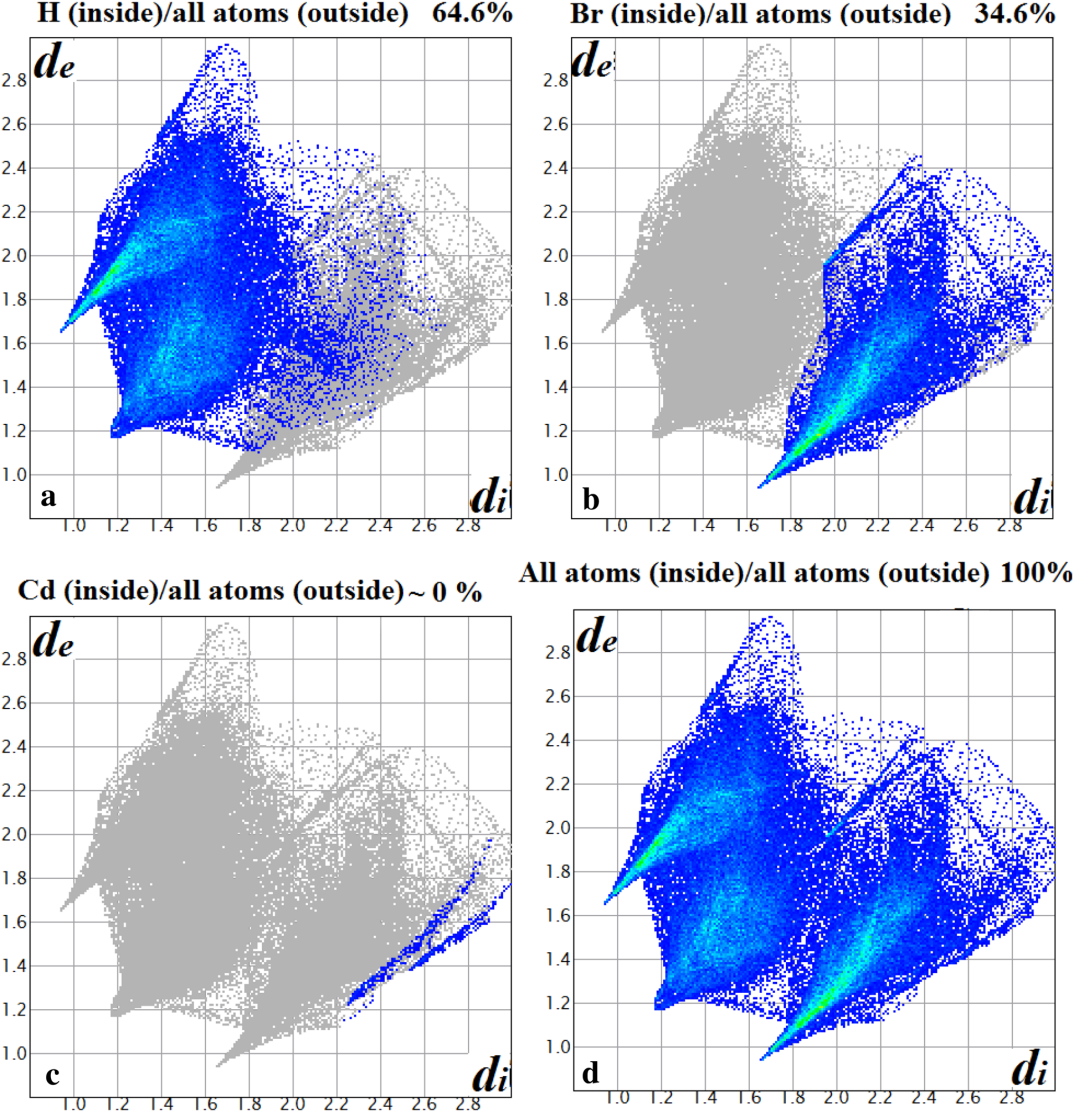
Hirshfeld surface fingerprint of complex **1**, **a** H_inside_…all atoms_outside_ 64.6 %, **b** Br_inside_…all atoms_outside_ 34.6 %, **c** Cd_inside_…all atoms_outside_ ~0 %, **d** all atoms_inside_…all atoms_outside_ 100 %, total interactions

The two-dimensional fingerprint plots over the Hirshfeld surfaces of complex **1** illustrate the significant differences between the intermolecular interaction patterns. H…all (64.6 %), Br…all (34.4 %), Cd…all (0.6 %) and all…all (Fig. 13) and Table 1.

**Table 1:** Inside/outside intermolecular interaction percentage by atoms

100 %	H_inside_	Br_inside_	Cd_inside_	N_inside_	C_inside_
H_outside_	41.7	32.7	0	0	0
Br_outside_	22.4	0.8	0	0	0
Cd_outside_	0.2	0	0	0	0
N_outside_	0	0	0	0	0
C_outside_	0	0	0	0	0

Table 1 illustrate the detail fingerprints intermolecular interaction between inside and outside atoms in both neighbor molecules.

## Experimental section

### Material and instrumentation

“Dien, dipn ligands and CdBr_2_•2.5H_2_O were purchased from Fluka. Elemental analyses were carried out on an ElementarVario EL analyzer. The IR spectra for samples were recorded using (Perkin Elmer Spectrum 1000 FT-IR Spectrometer). The UV–visible spectra were measured by using a TU-1901double-beam UV–visible spectrophotometer. TG/DTA spectra were measured by using a TGA-7 Perkin-Elmer thermogravimetric analyzer. The obtained nanoparticles were examined by a Bruker D/MAX 2500 X-ray diffractometer with Cu K radiation (λ = 1.54 Å), and the operation voltage and current were maintained at 40 kV and 250 mA, respectively. The transmission electron microscopy was (TEM, 1001 JEOL Japan). The scanning electron microscopy (SEM, JSM-6360 ASEM, JEOL Japan). The Hirshfeld surfaces analysis of complex **1** was carried out using the program CRYSTAL EXPLORER 3.1 [39]”.

### General procedure for the preparation of the desired complexes

In an ultrasonic open atmosphere media, a mixture of CdBr_2_•2.5H_2_O (2.0 mmol) in distilled ethanol (15 mL) and the free ligand was added in excess (6.0 mmol). The reaction mixture was subjected to ultrasonic vibration until the product complex appeared as white precipitate after ~20 min. The product was filtered and washed several times with ethanol. The product was only soluble in water, DMF and DMSO. Single crystals suitable for X-ray diffraction experiments were obtained by slow evaporation of water from complex solution.

#### Complex **1**

Yield: (91 %). Anal. Calc. for C_8_H_26_Br_4_Cd_2_N_6_: C, 12.80; H, 3.49; N, 11.19 %. *Found*. C, 12.53; H, 3.61; N, 11.28 %. MS [M^+2^] = 320.0 [theoretical = 320.2 m/z]. UV–Vis bands in water 275 nm. m.p 340 °C. Conductivity in DMF: 18.3 (µS/cm). ^1^H NMR (d^6^-DMSO): d (ppm) 2.55 and 2.62 (2br, 16H, 8CH_2_), 2.85 (br, 8H, 4NH_2_), 3.35 (br, 2H, 2NH), ^13^C{^1^H} NMR (d^6^-DMSO):d (ppm) 25.2 (s, 4C, CH_2_), 34.5 (s, 4C, CH_2_).

#### Complex **2**

Yield: (88 %). Anal. Calc. for C_12_H_34_Br_4_Cd_2_N_6_: C, 17.86; H, 4.25; N, 10.42 %. *Found*. C, 1**7.**48; H, 4.21; N, 10.38 %. MS [M^+2^] = 376.0 [theoretical = 376.19 m/z]. UV–Vis bands in water 285 nm. m.p 320 °C. Conductivity in DMF: 22.3 (µS/cm). ^1^H NMR (d^6^-DMSO): d (ppm) 1.85 (br, 8H, 4CH_2_), 2.62 and 2.82 (2 br, 16H, 8CH_2_), 2.88 (br, 8H, 4NH_2_), 3.38 (br, 2H, 2NH), ^13^C{^1^H} NMR (d^6^-DMSO):d (ppm) 20.0 (s, 4C, CH_2_), 25.8 (s, 4C, CH_2_), 34.9 (s, 4C, CH_2_).

### Crystallography

A colourless prism shaped single crystal of dimensions 0.35 × 0.23 × 0.19 mm of the title compound was chosen for an X-ray diffraction study. The X-ray intensity Data were collected on a Bruker APEX-II CCD area diffractometer and equipped with graphite monochromatic MoK_α_ radiation of wavelength 0.71073 Å at 100 (2) K. Cell refinement and data reduction were carried out using *SAINT PLUS* [24]. The structure was solved by direct methods and refined by full-matrix least squares method on *F*^2^ using *SHELXS* and *SHELXL* programs [40]. All the non-hydrogen atoms were revealed in the first difference Fourier map itself.All the hydrogen atoms were positioned geometrically and refined using a riding model. After ten cycles of refinement, the final difference Fourier map showed peaks of no chemical significance and the residuals saturated to 0.0237. The geometrical calculations were carried out using the program *PLATON* [41]. The molecular and packing diagrams were generated using the software *MERCURY* [42]. The details of the crystal structure and data refinement are given in Table 2. The list of bond lengths and bond angles of the non-hydrogen atoms are given in Table 3. Figure 6 represents the ORTEP of the molecule with thermal ellipsoids drawn at 50 % probability.

**Table 2:** Crystal data and structure refinement for Ligand and complex **1**

Parameter	Value
Empirical formula	C_8_H_26_Br_4_Cd_2_N_6_
Formula weight	750.79
Temperature	100 (2) K
Wavelength	0.71073 Å
Crystal system, space group	Monoclinic, *P21/n*
Unit cell dimensions	*a* = 9.4335 (12) Å *b* = 14.7512 (18) Å *c* = 14.7815 (18) Å β = 100.131 (2)°
Volume	2024.9 (4) Å^3^
Z, calculated density	4, 2.463 Mg/m^3^
Absorption coefficient	9.993 mm^−1^
*F*_*(000)*_	1408
Crystal size	0.35 × 0.23 × 0.19 mm
Theta range for data collection	1.97–28.28°
Limiting indices	−12≤ h ≤12, 0≤ k ≤19, 0≤l≤19
Reflections collected/unique	4969/4960 [R(int) = 0.0000]
Refinement method	Full-matrix least-squares on *F* ^*2*^
Data/restraints/parameters	4969/0/181
Goodness-of-fit on *F* ^*2*^	1.057
Final R indices [*I* >2σ(*I*)]	*R1* = 0.0237, *wR2* = 0.0468
R indices (all data)	*R1* = 0.0328, *wR2* = 0.0494
Largest diff. peak and hole	0.595 and −0.885 e. Å^−3^

**Table 3:** Selected bond distances (Å) and bond angles (°) of complex **1**

Atoms	Length	Atoms	Length
Cd1-N14	2.346 (2)	C12-N13	1.472 (4)
Cd1-N20	2.357 (2)	N14-C15	1.475 (4)
Cd1-N7	2.365 (3)	C15-C16	1.516 (4)
Cd1-N13	2.365 (3)	C16-N17	1.469 (4)
Cd1-N17	2.410 (2)	N17-C18	1.471 (4)
Cd1-N10	2.422 (3)	C18-C19	1.512 (4)
N7-C8	1.474 (4)	C19-N20	1.476 (4)
C8-C9	1.517 (5)	Cd2-Br5	2.5721 (5)
C9-N10	1.463 (4)	Cd2-Br4	2.5809 (5)
N10-C11	1.468 (4)	Cd2-Br6	2.5835 (4)
C11-C12	1.514 (5)	Cd2-Br3	2.6313 (5)

Atoms	Angle	Atoms	Angle
N14-Cd1-N20	141.05 (9)	C11-N10-Cd1	107.43 (19)
N14-Cd1-N7	88.75 (9)	N10-C11-C12	109.8 (3)
N20-Cd1-N7	90.10 (9)	N13-C12-C11	110.7 (3)
N14-Cd1-N13	91.91 (9)	C12-N13-Cd1	111.76 (19)
N20-Cd1-N13	111.72 (9)	C15-N14-Cd1	108.88 (18)
N7-Cd1-N13	142.31 (9)	N14-C15-C16	109.2 (3)
N14-Cd1-N17	74.73 (8)	N17-C16-C15	110.1 (3)
N20-Cd1-N17	74.29 (9)	C16-N17-C18	114.7 (2)
N7-Cd1-N17	125.05 (9)	C16-N17-Cd1	107.94 (18)
N13-Cd1-N17	91.21 (9)	C18-N17-Cd1	107.01 (18)
N14-Cd1-N10	121.49 (9)	N17-C18-C19	109.7 (3)
N20-Cd1-N10	95.39 (9)	N20-C19-C18	109.4 (3)
N7-Cd1-N10	73.99 (9)	C19-N20-Cd1	110.46 (18)
N13-Cd1-N10	73.68 (9)	Br5-Cd2-Br4	109.305 (14)
N17-Cd1-N10	157.38 (9)	Br5-Cd2-Br6	108.258 (14)
C8-N7-Cd1	110.03 (19)	Br4-Cd2-Br6	111.585 (14)
N7-C8-C9	109.8 (3)	Br5-Cd2-Br3	111.083 (13)
N10-C9-C8	110.6 (3)	Br4-Cd2-Br3	104.874 (13)
C9-N10-C11	114.8 (3)	Br6-Cd2-Br3	111.720 (16)
C9-N10-Cd1	108.82 (19)		

## Conclusions

For the first time, two new complexes [Cd(dien)_2_]CdBr_4_ and [Cd(dipn)_2_]CdBr_4_ were synthesized in good yield. The chemical structure of [Cd(dien)_2_]CdBr_4_ was confirmed by X-ray diffraction. The Cd(II) cation center are located in a slightly distorted octahedral geometry while Cd(IV) anion are in tetrahedral and in high stability. Thermolysis of the complexes revealed the formation of CdO cubic nanoparticle, which was deduced by XRD, FT-IR, TEM and SEM, the average size of CdO nanoparticles found to be 60 nm.

## Supplementary material

Crystallographic data for complex **1** has been deposited with the Cambridge Crystallographic Data Centre as supplementary publication number CCDC 1404033. “Copies of this information may be obtained free of charge via http://www.ccdc.cam.ac.uk/conts/retrieving.html (or from the CCDC, 12 Union Road, Cambridge CB2 1EZ, UK; fax: +44-1223-336033; e-mail: deposit@ccdc.cam.ac.uk)”.
